# Supplementary material for: Oxidation of p-[125I]Iodobenzoic Acid and p-[211At]Astatobenzoic Acid Derivatives and Evaluation In Vivo
Source: Int J Mol Sci. 2022 Sep 13;23(18):10655. doi: 10.3390/ijms231810655 (PMC9506049; doi:10.3390/ijms231810655)
Supplement: Supplementary file 1 [file ijms-23-10655-s001.zip › ijms-1843644-supplementary.pdf]

Supplementary Materials

for

Oxidation of *p*-[^{125}I]iodobenzoic acid and *p*-[^{211}At]astatobenzoic acid derivatives and evaluation in vivo

Table S1: Concentration (%ID/g) of Radioactivity for Co-injected O_2X-Ph-dPEG_4-CO_2Me derivatives [^{125}I]11 and [^{211}At]13 in selected tissues of athymic mice.[a]

Tissues	1 h[b,c]		4 h		24h	
	I-125	At-211	I-125	At-211	I-125	At-211
blood	0.22 ± 0.14	1.19 ± 0.20	0.23 ± 0.18	1.05 ± 0.11	0.01 ± 0.00	0.05 ± 0.03
muscle	0.12 ± 0.05	0.65 ± 0.15	0.03 ± 0.01	0.54 ± 0.04	0.00 ± 0.00	0.01 ± 0.01
lung	0.32 ± 0.10	7.73 ± 1.52	0.14 ± 0.07	6.60 ± 0.55	0.54 ± 0.85	1.65 ± 1.41
kidney	0.84 ± 0.23	3.10 ± 0.61	0.40 ± 0.15	2.65 ± 0.18	0.03 ± 0.01	0.24 ± 0.08
spleen	0.35 ± 0.28	8.81 ± 6.44	0.08 ± 0.04	8.09 ± 0.62	0.01 ± 0.00	0.38 ± 0.31
liver	1.73 ± 0.96	1.19 ± 0.23	0.64 ± 0.47	1.05 ± 0.12	0.01 ± 0.00	0.11 ± 0.03
intestine	9.59 ± 8.73	2.29 ± 0.77	3.39 ± 1.15	1.70 ± 0.29	0.02 ± 0.01	0.19 ± 0.06
neck	0.49 ± 0.09	10.43 ± 2.77	0.35 ± 0.14	11.78 ± 4.88	0.02 ± 0.01	2.52 ± 1.57
stomach	3.53 ± 1.09	19.24 ± 6.82	0.65 ± 0.16	20.75 ± 5.99	0.02 ± 0.01	1.69 ± 0.49

[a]Values shown are % injected dose / gram ± standard deviation. [b]Time of sacrifice from coinjection of [^{125}I]**11** and [^{211}At]**13**. [c]Data were obtained for n = 5 mice at each time point; average animal weight for the 3 time-groups, 26.43 ± 1.83 g; Injectate for each animal had 3 μCi of [^{125}I]**11** and 3 μCi of [^{211}At]**13** in approximately 100 μL of 0.9% sterile saline.

Table S2: Concentration (%ID/g) of Radioactivity for Co-injected [^{125}I]$NaIO_3$ and [^{211}At]$NaAtO_3$ in selected tissues of athymic mice.[a]

Tissues	1 h[b,c]		4 h		24h	
	I-125	At-211	I-125	At-211	I-125	At-211
blood	2.15 ± 0.36	1.23 ± 0.10	1.55 ± 0.37	1.12 ± 0.09	0.02 ± 0.00	0.03 ± 0.06
muscle	0.57 ± 0.05	0.60 ± 0.09	0.36 ± 0.09	0.45 ± 0.06	0.01 ± 0.00	0.00 ± 0.06
lung	1.82 ± 0.40	7.05 ± 1.26	1.23 ± 0.28	5.78 ± 0.78	0.05 ± 0.02	1.03 ± 0.53
kidney	1.48 ± 0.36	3.02 ± 0.46	1.17 ± 0.37	2.74 ± 0.24	0.04 ± 0.01	0.33 ± 0.22
spleen	1.14 ± 0.22	6.79 ± 1.46	0.98 ± 0.54	6.37 ± 1.05	0.02 ± 0.00	0.49 ± 0.25
liver	0.84 ± 0.15	1.06 ± 0.11	0.73 ± 0.18	1.08 ± 0.16	0.03 ± 0.01	0.16 ± 0.07
intestine	1.08 ± 0.30	1.49 ± 0.09	0.78 ± 0.27	1.56 ± 0.22	0.02 ± 0.01	0.23 ± 0.12
neck	10.71 ± 2.39	9.74 ± 2.42	11.78 ± 3.26	13.56 ± 4.81	0.39 ± 0.33	4.30 ± 3.18
stomach	13.52 ± 3.27	17.35 ± 4.81	8.61 ± 2.69	17.50 ± 5.33	0.13 ± 0.05	2.04 ± 0.92

[a]Values shown are % injected dose / gram ± standard deviation. [b]Time of sacrifice from coinjection of [^{125}I]$NaIO_3$ and [^{211}At]$NaAtO_3$. [c]Data were obtained for n = 5 mice at each time point; average animal weight: 1 h group; 31.30 ± 1.71 g; 4 h group; 32.42 ± 1.42 g; 24 h group; 22.92 ± 1.93 g. Injectate for each animal had ~5 μCi of [^{125}I]$NaIO_3$ and ~5 μCi of [^{211}At]$NaAtO_3$ in approximately 100 μL of 0.9% sterile saline.
